# Mammalian prion protein (PrP) forms conformationally different amyloid intracellular aggregates in bacteria

**DOI:** 10.1186/s12934-015-0361-y

**Published:** 2015-11-04

**Authors:** Bruno Macedo, Ricardo Sant’Anna, Susanna Navarro, Yraima Cordeiro, Salvador Ventura

**Affiliations:** Faculdade de Farmácia, Centro de Ciências da Saúde, Universidade Federal do Rio de Janeiro, Av. Carlos Chagas Filho 373, Bloco B, Subsolo, Sala 17, Rio de Janeiro, RJ 21941-902 Brazil; Institut de Biotecnologia i de Biomedicina, Universitat Autònoma de Barcelona, 08193 Bellaterra, Spain; CAPES Foundation, Ministry of Education of Brazil, Brasilia, DF 70040-020 Brazil; Departament de Bioquímica i Biologia Molecular, Facultat de Biociències, Universitat Autònoma de Barcelona, 08193 Bellaterra, Spain

**Keywords:** Mammalian prions, Protein aggregation, Protein conformation, Inclusion bodies, Amyloids, *E. coli*

## Abstract

**Background:**

An increasing number of proteins are being shown to assemble into amyloid structures that lead to pathological states. Among them, mammalian prions outstand due to their ability to transmit the pathogenic conformation, becoming thus infectious. The structural conversion of the cellular prion protein (PrP^C^), into its misfolded pathogenic form (PrP^Sc^) is the central event of prion-driven pathologies. The study of the structural properties of intracellular amyloid aggregates in general and of prion-like ones in particular is a challenging task. In this context, the evidence that the inclusion bodies formed by amyloid proteins in bacteria display amyloid-like structural and functional properties make them a privileged system to model intracellular amyloid aggregation.

**Results:**

Here we provide the first demonstration that recombinant murine PrP and its C-terminal domain (90–231) attain amyloid conformations inside bacteria. Moreover, the inclusions formed by these two PrP proteins display conformational diversity, since they differ in fibril morphology, binding affinity to amyloid dyes, stability, resistance to proteinase K digestion and neurotoxicity.

**Conclusions:**

Overall, our results suggest that modelling PrP amyloid formation in microbial cell factories might open an avenue for a better understanding of the structural features modulating the pathogenic impact of this intriguing protein.

**Electronic supplementary material:**

The online version of this article (doi:10.1186/s12934-015-0361-y) contains supplementary material, which is available to authorized users.

## Background

Protein aggregation is the hallmark of many neurodegenerative diseases, including Alzheimer’s (AD), Parkinson’s (PD), and the Transmissible Spongiform Encephalopathies (TSEs) [1], also termed prion diseases. The misfolding of a particular protein, i.e., the β-amyloid peptide (Aβ) for AD, α-synuclein (α-syn) for PD, and prion protein (PrP) for TSEs can lead to its abnormal accumulation in tissues, which usually comes along with severe cellular damages. Irrespectively of the misfolded protein sequence and structure, protein aggregation usually proceeds in a well-organized fashion to form amyloids in these diseases [1]. Amyloid fibrils architecture is characterized by a β-sheet enriched core, which usually binds to Congo red (CR) and thioflavin-T (Th-T) dyes [2].

**Fig. 1:**
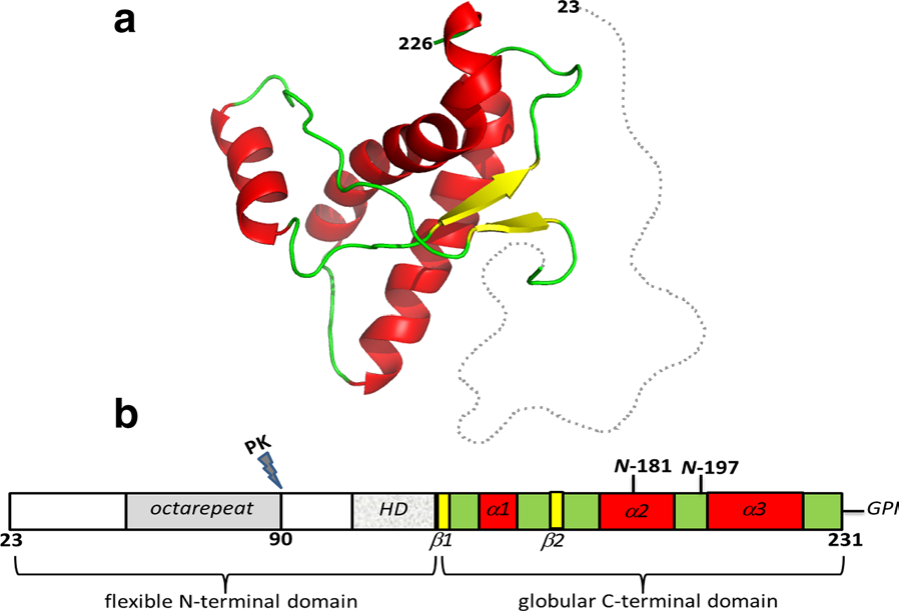
Mouse PrP structure and domain organization. **a** Three dimensional structure of the globular domain of mouse PrP (only residues 124–226 are assigned by NMR), PDB: 1AG2. Alpha helices 1, 2 and 3 are represented in *red*; beta strands 1 and 2 in *yellow*; loops and turns in *green*. The *dotted gray line* represents the unstructured N-terminal domain, comprising residues 23–120 (figure done with PyMol and disordered domain drawn with Inkscape). **b** Scheme of the primary structure of mature murine PrP (residues 23–231). *HD* hydrophobic domain; β-sheets are depicted in *yellow*, α-helices in *red*, and the remaining of the C-terminal domain in *gree*n; N-181 and N-197 are sites of glycosylation; *GPI* glycosylphosphatidylinositol anchor; *PK* proteinase K cleavage site

TSEs form a group of lethal neurodegenerative disorders, which affect both humans and other mammals [3]. They may manifest as infectious, genetic or sporadic diseases. The structural conversion of the cellular prion protein (PrP^C^), into its misfolded pathogenic form (PrP^Sc^) is the central event of these pathologies. PrP^C^, which is found anchored to the extracellular membrane of several cell types, has a well-defined structure, with a highly flexible and unstructured N-terminal tail and a globular C-terminal domain composed by three α-helices and two short antiparallel β-strands [4, 5] (Fig. 1). Unlike PrP^C^, PrP^Sc^ is an insoluble protein, mainly composed by β-sheet structures, partially resistant to proteolysis, with a high propensity to form both amorphous and amyloid-like aggregates [3, 6, 7]. Deposition of aggregated PrP^Sc^ in tissues is attributed to cause neurodegeneration. PrP^Sc^ aggregation becomes self-perpetuating in vivo through the conversion of host PrP^C^ into abnormal PrP^Sc^, in a process catalyzed by the infectious form [6, 7]. In vitro, the assembly of prions into amyloids displays a typical nucleation-elongation reaction, but in the presence of preformed fibrillar seeds the ‘lag-phase’, corresponding to the nucleation reaction, is abrogated. The reduction in the lag-phase evidences the ability of the seed to catalyze amyloid polymerization [7–9], a property that underlies the mechanism of prion conformational replication [10]. It is assumed that the PrP can adopt multiple misfolded conformations that are the molecular origin of prion strains and dictate the efficiency of the species barrier in the transmission of prions [11]. Distinct prion strains can lead to phenotypically different prion diseases in animals, with different incubation times and brain deposition profiles [12–14].

Inclusion bodies (IBs) formation in bacteria has long been regarded as an unspecific process resulting from the collapse of hydrophobic contacts between partially or totally unfolded species after protein synthesis at the ribosome [15]. However, an increasing body of evidence indicates that the bacterial IBs formed by amyloidogenic proteins share a number of common structural features with the highly ordered and, in many cases, pathogenic amyloid fibrils [16–19]. Interestingly, it was shown that a specific domain of a bacterial DNA replication protein, the RepA-WH1, assembles into fibrils and, when expressed in *E. coli*, can lead to a peculiar amyloidosis through the inhibition of bacterial proliferation [19]. These RepA-WH1 aggregated particles can be vertically transmitted across generations, thus this protein was characterized as a synthetic bacterial prionoid [19]. Therefore, bacteria have become a simple model system to study intracellular protein aggregation under biologically relevant conditions that cannot be easily recapitulated in vitro, such as the presence of chaperones and proteases, molecular crowding, and the continuous synthesis of the protein in the ribosome [20–22].

Het-s, from the fungus *Podospora anserina*, was the first prion protein whose bacterial IBs were shown to display amyloid-like properties [23, 24]. The differential trait of these aggregates emerged when they were transfected into prion-free fungal strains, as they promoted prionic conversion [23]. This result was later corroborated for the yeast prion Sup35 [25, 26]. The amyloid-like IBs of Sup35 induced the prion phenotype in prion-free yeast strains, the infectivity rate being modulated by the environmental conditions during the formation of IBs [25–27]. These observations provide perhaps the best evidence that the IBs molecular structure can recapitulate the architecture of amyloid fibrils, in such a way that even the infectious properties of amyloids, which depend on specific conformational features, seem to be conserved in the two type of aggregates.

It was previously shown that bacterially expressed recombinant murine PrP can be turned infectious in vitro causing prion pathology when inoculated in mice [28]. Here, we address whether, like their fungal counterparts, mammalian PrP can form amyloid intracellular aggregates when expressed in bacteria. With this aim, we produced, purified and conformationally characterized the intracellular aggregates formed by the wild-type murine PrP encompassing residues 23–231 (PrP^WT^) and the C-terminal domain of murine PrP (PrP^90–231^) (Fig. 1). Our current study provides the first demonstration that recombinant murine PrPs can form amyloid structures inside bacterial IBs. Besides, although possessing similar secondary structure, PrP^WT^ IBs and PrP^90–231^ IBs exhibit conformational diversity, as they bind CR and Th-T dyes to different extents, display distinct morphology, different stability and resistance against proteinase K proteolysis. These conformational differences result in different toxicity of the two PrP IBs resistant cores when added to neuroblastoma cells in culture.

## Results and discussion

### Aggregation of PrP^WT^ and PrP^90–231^ into IBs in bacteria

The inherent aggregation propensity of amyloid proteins often results in their aggregation into insoluble IBs when they are produced in bacteria [29]. In several cases, these intracellular aggregates have been shown to display amyloid-like properties. To verify if this is the case for mammalian prion proteins, we expressed the murine wild-type prion protein (PrP^WT^) encompassing residues 23-231 (PrP^WT^) and the C-terminal domain of murine PrP (PrP^90–231^) in bacteria and purified the resulting IBs. Both PrP forms [either the full-length, mature PrP (PrP^WT^) or the truncated fragment 90–231] can exist in vivo in healthy and diseased brain and have been extensively studied [30–32]. The N-terminal unstructured domain is proposed to participate in PrP physiological function because of its ability to bind to different classes of partners, including copper ions (Cu^2+^), glycosaminoglycans (GAGs), nucleic acids (NAs) and lipids [33–38]. It is proposed that PrP^C^ acts as cell surface scaffold protein, gathering different partners in a macromolecular assembly to participate in cell signalling [39]. PrP^C^ undergoes endoproteolytic attack within its N-terminal domain, leading to the appearance of C-terminal fragments attached to the plasma membrane and soluble N-terminal peptides [30]. Both in normal and pathological brains one of these cleavages occurs at position 90, thereby generating PrP^90–231^ C-terminal fragment. The truncated PrP encompassing residues 90–231 corresponds to the proteinase K-resistant core of the pathogenic PrP^Sc^, referred also as PrP^Res^ [30]. Although the N-terminal domain appears to be unnecessary for prion propagation, since the fragment PrP^Res^ is capable of transmitting prion disease in vivo [40]; this region may affect the pathways of prion misfolding and substantially impact PrP^Sc^ conformational diversity. Furthermore, in vivo studies have shown that mice expressing N-terminally truncated PrP develop disease more slowly and are less susceptible to infection than mice expressing PrP^WT^ [41].

**Fig. 2:**
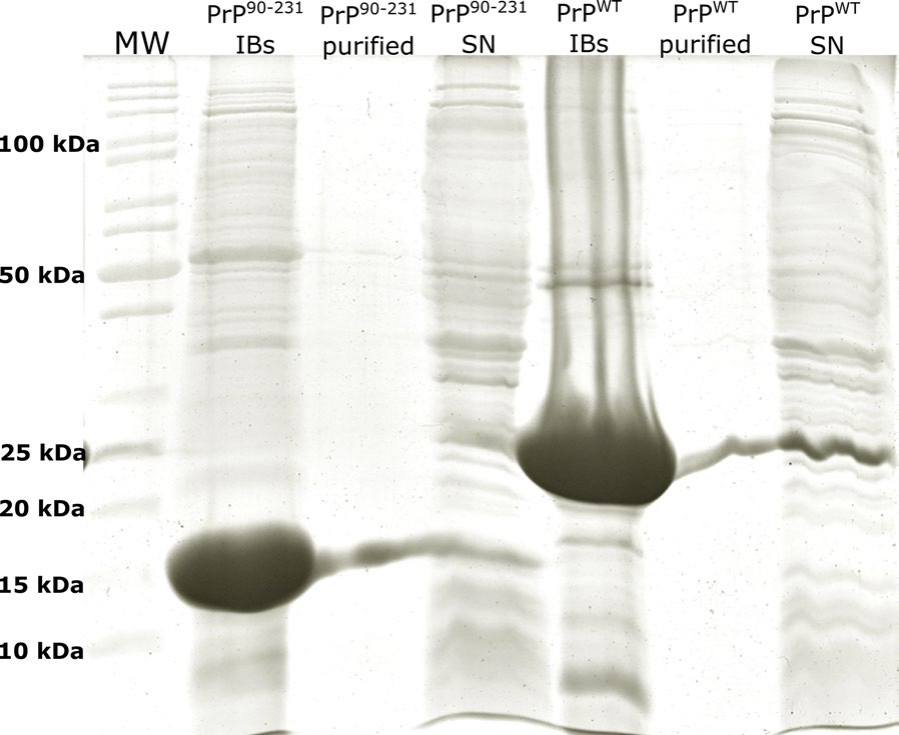
Isolation of PrP^WT^ and PrP^90−231^ IBs from *E. coli* cells. SDS-PAGE analysis of purified PrPs IBs. The band at ~16 kDa corresponds to the C-terminal region of PrP (PrP^90−231^) and that at ~25 kDa to the PrP^WT^ (rPrP^23−231^), both with the histidine-tag. The purification of both PrP monomers from their respective isolated IBs is also shown, as well as the remaining soluble proteins present in the supernatant after cell fractionation (SN). Molecular weight markers are shown on the *left* (MW)

PrP forms were expressed essentially as insoluble proteins in bacteria, with only a small portion of the protein residing in the soluble fraction. Accordingly, IBs were purified from the insoluble cellular fraction (Fig. 2). PrPs were the major component in purified IBs, with PrP^WT^ displaying a molecular weight of ~25 kDa and the PrP^90–231^ between 15 and 20 kDa, according to respective bands in SDS-PAGE (Fig. 2). The approximate molecular weights obtained from the electrophoresis gel are in accordance with the theoretical molecular masses for both PrP^WT^ and PrP^90−231^ (23 and 16 kDa, respectively), with the addition of the histidine-tag (+~3 kDa) (see “Methods”). Further refolding, purification and cleavage steps allowed extraction of soluble and pure PrP from the IBs (see “Methods”) for subsequent in vitro fibril formation and seeding analysis.

### PrP^WT^ and PrP^90–231^ form β-sheet enriched IBs

The aggregation of proteins into amyloid fibrils results in the formation of intermolecular β-sheets [16, 42]. Attenuated Total Reflectance–Fourier Transform Infrared spectroscopy (ATR-FTIR) permits addressing the structural characteristics of protein aggregates [43–46].

**Fig. 3:**
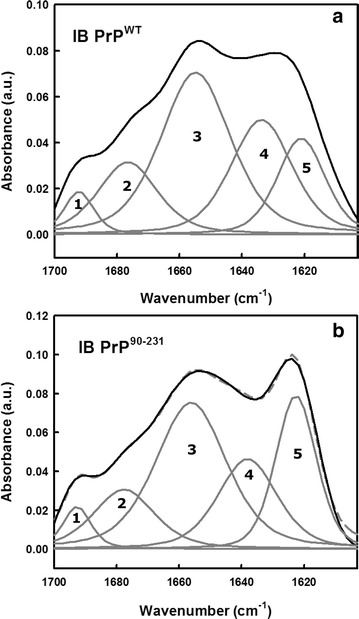
Secondary structure analysis of PrPs IBs by ATR-FTIR. The secondary structure content of PrP^WT^ (**a**) and PrP^90−231^ (**b**) inside the IBs was determined following ATR-FTIR absorbance of dry samples in the amide I region of the infrared spectrum (*solid black line*). Spectral components in the Fourier deconvoluted FTIR spectra (*dashed gray line*) are shown as *solid gray lines* (bands 1–5). The area and position of the correspondent bands are indicated in Table 1

To get insights into the secondary structure content of the two purified PrP IBs, we analysed the amide I region of the FTIR spectrum (1700–1600 cm^−1^) (Fig. 3). This region corresponds to the absorption of the carbonyl peptide bond group of the protein main chain and is a sensitive marker of the protein secondary structure [47]. Deconvolution of the ATR-FTIR-absorbance spectra of PrP^WT^ and PrP^90–231^ IBs allows us to assign the individual secondary structure elements and their relative contribution to the main absorbance signal (Fig. 3; Table 1). In contrast to soluble purified PrPs, which exhibit a predominant α-helical secondary structure content (Additional file 1), as previously assessed by FTIR and circular dichroism [36, 48, 49], it is evident that the truncated form (PrP^90–231^) has a higher contribution of β-sheets (Fig. 3; Table 1). The low frequency peak at ~1620 cm^−1^ together with the low intensity, high frequency band at ~1690 cm^−1^ (band 1, Fig. 3a, b) are attributed to antiparallel beta-sheets found in fibrils [46]. In addition, the peak at 1634–1638 cm^−1^ (band 4, Fig. 3a, b) found in both IBs is assigned to parallel β-sheets, indicating a mixed β-sheet composition for PrPs found in inclusion bodies. In general, the observed absorption FTIR spectra is similar to the one shown for prion rods extracted from scrapie-infected hamster brains [50]. As also seen for infectious prion rods [50], part of the native structure was maintained for both extracted IBs (peaks at ~1655 cm^−1^ attributed to α-helical/disordered structure). Thus, we suggest that a fraction of the molecules are adopting the intrinsic native helical fold, since native protein structure has been described to be retained in the IBs formed by certain proteins, such as GFP and others [51, 52].

**Table 1:** Assignment of secondary structure components of purified *E. coli* PrP^WT^ and PrP^90−231^ IBs in the amide I region of ATR-FTIR spectra

PrP^WT^ IBs			PrP^90−231^ IBs		
Band (cm^−1^)	Area (%)	Secondary structure	Band (cm^−1^)	Area (%)	Secondary structure
1621	16	β-Sheet	1623	23	β-Sheet
1634	25	β-Sheet	1638	20	β-Sheet
1655	40	Unordered/α-helix	1656	39	Unordered/α-helix
1677	15	β-Sheet/turns	1678	14	β-Sheet/turns
1692	4	β-Sheet	1693	4.5	β-Sheet

Amide I ATR-FTIR spectra deconvolution and band assignment was done as described in the Methods section with OMNIC™ software. Band frequencies deviation: ±4 cm^−1^. The depicted wavenumbers refer to bands 1–5 (from the higher to the lower frequency) obtained from Fig. 3.

### Stability of PrPs IBs towards chemical denaturation

**Fig. 4:**
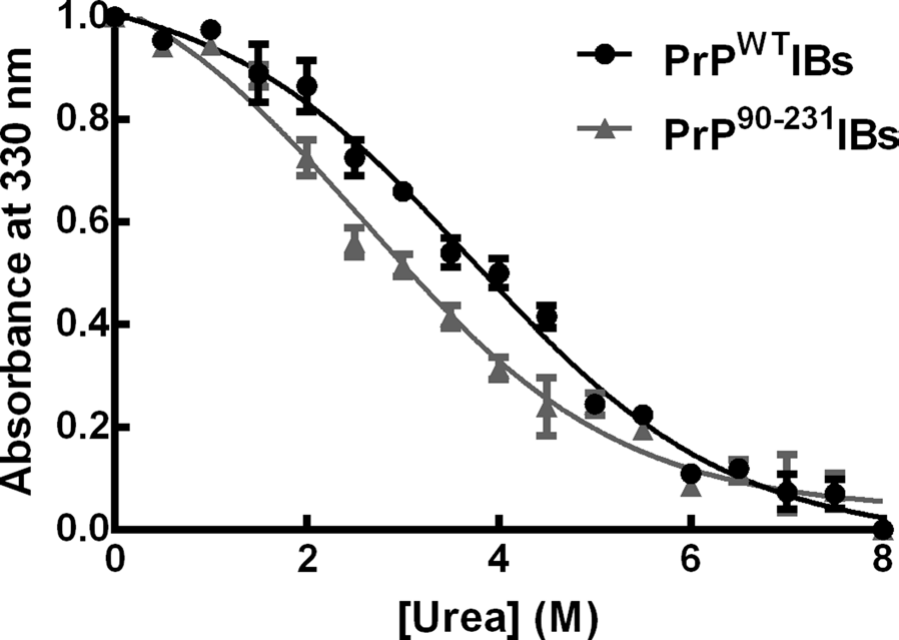
PrPs IBs present different stability against urea treatment. Solubilization of PrPs IBs (OD_350_ = 1.0) at equilibrium in the presence of increasing concentrations of urea, monitored by light scattering at 350 nm at room temperature. Values shown are the mean ± SD

The presence of regular β-sheet secondary structure inside PrPs amyloid-like IBs implies the existence of cooperative interactions between the polypeptide chains embedded in these aggregates. To confirm this assumption, we used chemical denaturation with urea. We have shown before that this approach allows approximating the conformational stability of bacterial intracellular aggregates [53]. IBs solubilization was measured by monitoring the changes in absorbance at 350 nm in urea concentrations ranging from 0 to 8 M. The cooperative denaturation transitions observed for both PrPs IBs support the presence of selective contacts inside these aggregates (Fig. 4). We calculated [urea]_1/2_ for IBs solubilization to be 3.72 ± 0.10 and 2.61 ± 0.20 M, for PrP^WT^ and PrP^90–231^IBs, respectively; exhibiting thus significantly different conformational stability. The stability of these in vivo formed PrP aggregates is, however, lower than the one reported for in vitro formed PrP^WT^ fibrils, with m_1/2_ ~ 4.5 M in guanidine hydrochloride-induced denaturation experiments [54]. This is not surprising if we take into account that ordered and disordered conformations appear to coexist in IBs and that the presence of minor concentrations of other proteins might also condition the stability of these in vivo formed aggregates.

### PrP^WT^ and PrP^90–231^ IBs bind to thioflavin-S in living cells

**Fig. 5:**
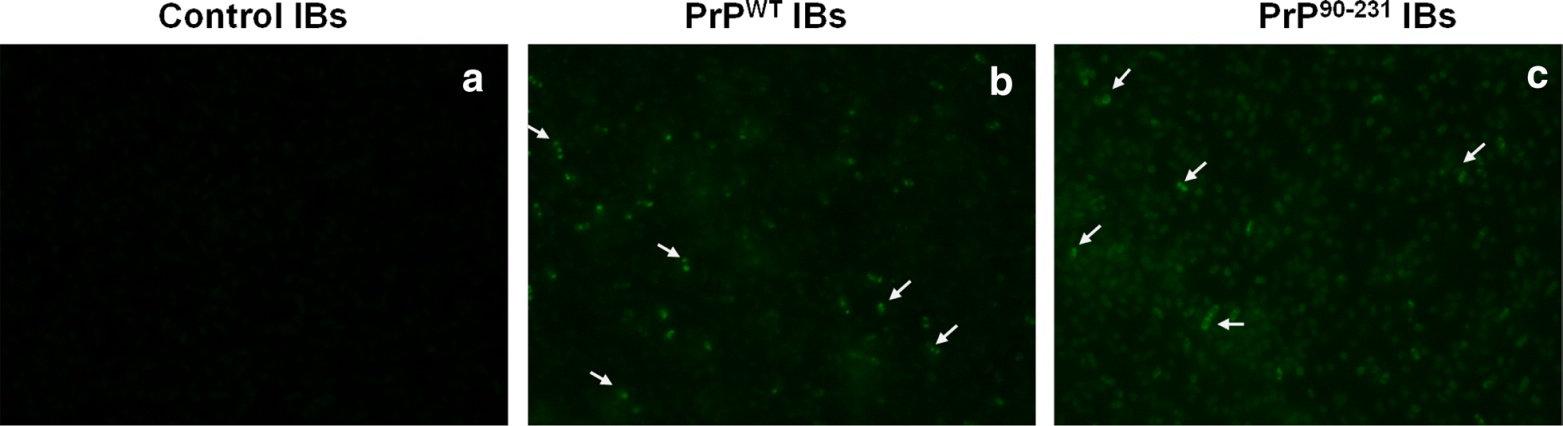
Th-S staining in intact bacterial cells expressing PrPs. Bacteria expressing recombinant PrPs (**b** PrP^WT^; **c** PrP^90–231^) or containing an empty plasmid (control) (**a**) were stained with Th-S and observed at ×40 magnification by fluorescence microscopy to visualize the *green* fluorescence characteristic of amyloid intracellular structures. *Arrows* indicate the position of IBs

We have shown recently that thioflavin-S (Th-S) staining of living bacterial cells can be used to detect the presence of intracellular amyloid-like structures as well as to find inhibitors that interfere with amyloid formation [55, 56]. The staining of cells expressing PrP^WT^ and PrP^90–231^ was monitored using fluorescence microscopy. As it can be observed in Fig. 5, induced cells exhibited a green fluorescent background with fluorescent foci located at the cell poles, suggesting that these proteins adopt amyloid-like conformation in bacterial IBs. In contrast, induced cells containing an empty plasmid exhibited only residual fluorescence.

### Amyloid properties of PrP^WT^ and PrP^90–231^ IBs

We evaluated the binding of purified PrP IBs to the amyloid diagnostic dyes CR and Th-T to confirm that the prevalent β-sheet in these aggregates has an amyloid-like nature and to further explore if the IBs would present different amyloid properties. To evaluate the specific contribution of PrP IBs in these assays, relative to that of other proteins possibly present in this fraction, cells bearing the same plasmid without any insert were induced and the insoluble fraction purified in the same manner than those containing the PrP cDNAs and used as negative control.

**Fig. 6:**
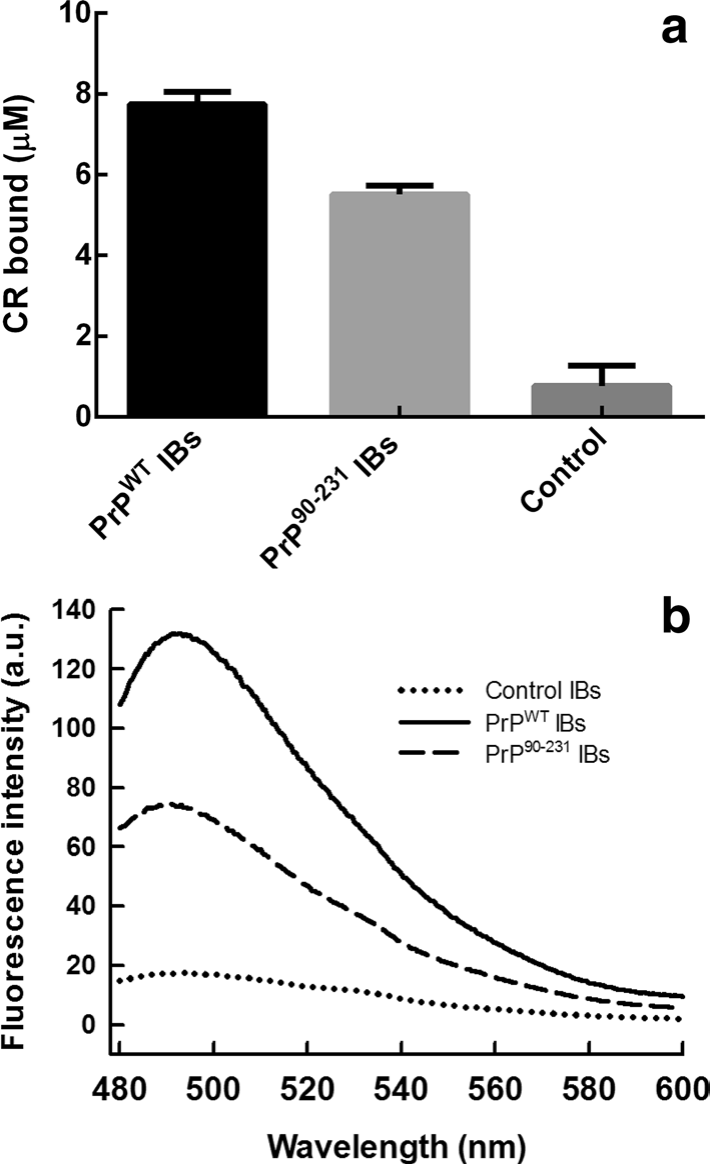
Specific binding of amyloid dyes to PrPs IBs. **a** CR binding assay in the presence of PrPs IBs and control insoluble fraction (OD_350_ = 1.0); the extent of binding is calculated as described in “Methods”. **b** Fluorescence emission spectrum of Th-T in the presence of PrPs IBs and control insoluble fraction (OD_350_ = 0.1). Samples were excited at 445 nm and emission was collected from 480 to 580 nm. Both assays were done in PBS

The absorbance of CR increases and the spectrum maximum red-shifts in the presence of amyloid-like structures [15]. We calculated the amount of CR bound in relation to the negative control (see “Methods”). We observed a ~tenfold increase for PrP^WT^ IBs and sevenfold increase for PrP^90–231^ IBs in relation to the control insoluble fraction (Fig. 6a).

ThT fluorescence emission increases significantly when the dye binds to amyloid fibrils [57]. Both PrPs IBs promote a strong increase in Th-T fluorescence (Fig. 6b). In agreement with CR data, PrP^WT^ IBs promote a larger increase in Th-T fluorescence (eightfold) than the promoted by PrP^90–231^ IBs (3.5-fold), whereas the negative control did not induce any significant increase in fluorescence emission relative to free Th-T (Fig. 6b). Although these two PrP IBs possesses similar secondary structure content, these data indicate that murine PrP^WT^ and PrP^90–231^ adopt amyloid-like structures when they aggregate intracellularly in bacteria, displaying, however, different fibrillar structures. Indeed, it is known that mature fibrils of different PrP species, like hamster or mice, have similar secondary structures but show variation in fibrillar morphology [58].

### Amyloid morphology of PrPs IBs

**Fig. 7:**
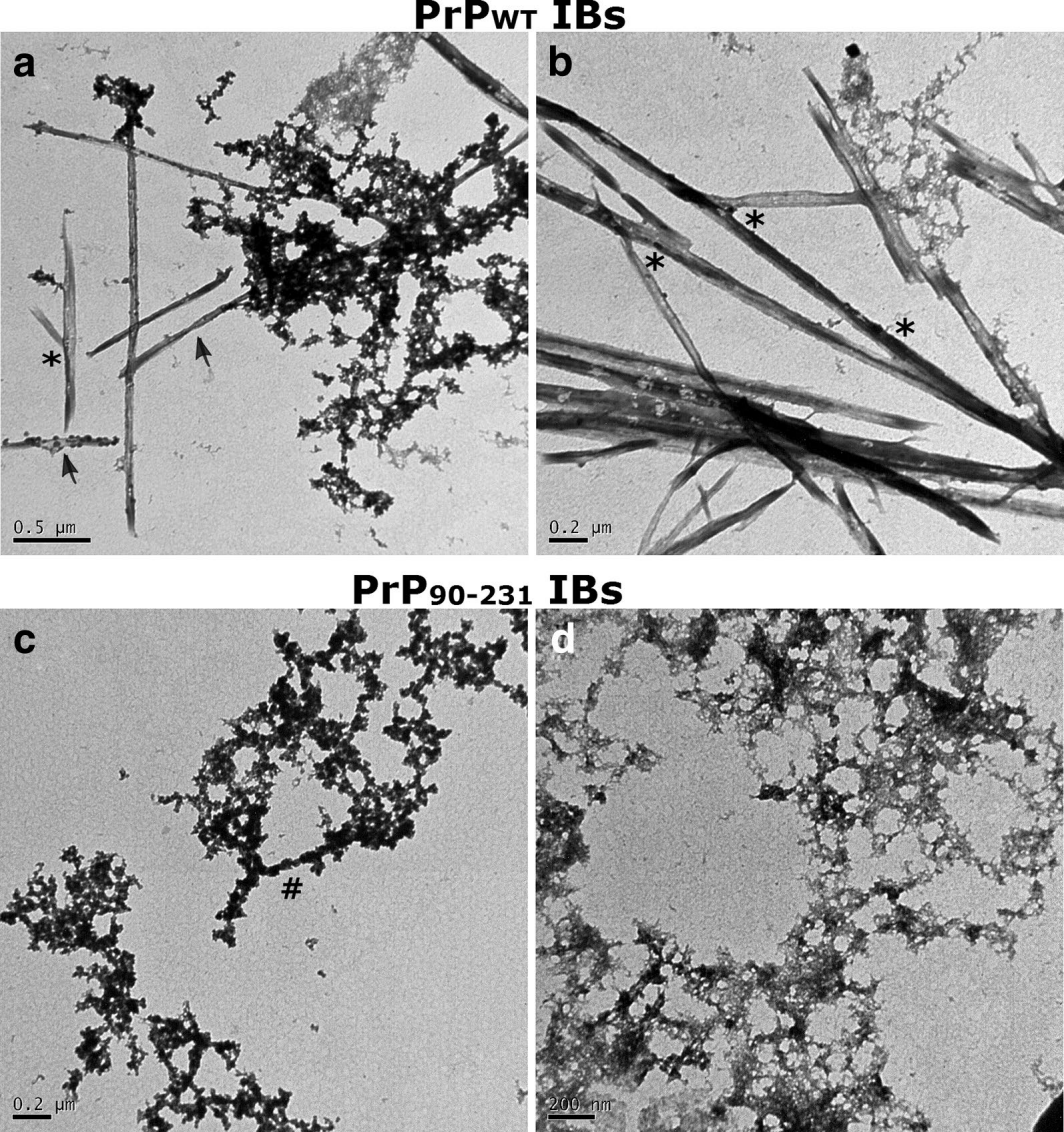
Morphological analysis of PrPs inside IBs. Fresh and intact PrP^WT^ (**a**, **b**) and PrP^90–231^ (**c**, **d**) IBs observed by negative staining and transmission electron microscopy display amyloid-like structures. *Scale bars* 0.5 μm (**a**), 0.2 μm (**b**), 0.2 μm (**c**, **d**). The *arrows* indicate single filaments; the *asterisks* mean unzipped filaments and the *hash* indicates the initial step of a protofibril formation

**Fig. 8:**
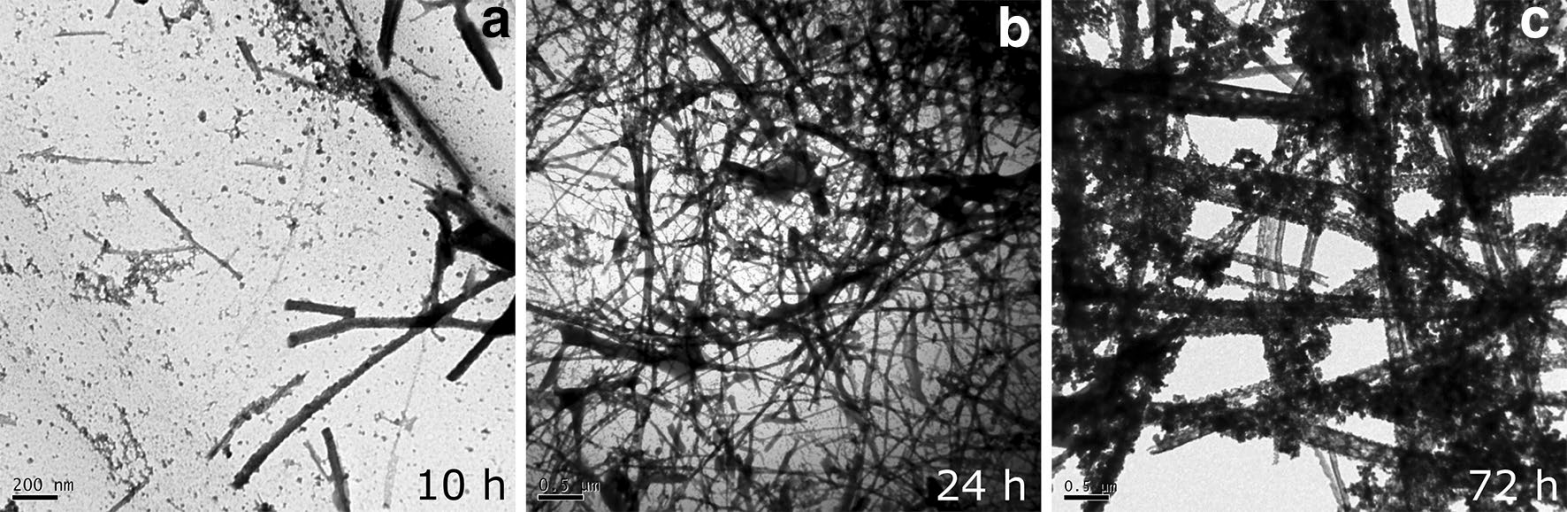
Evolution of PrP^WT^ aggregates morphology during in vitro aggregation. After purification and refolding PrP^WT^ was converted into amyloid fibrils as described in “Methods”. The aggregates morphology was assessed at different times by negative staining and transmission electron microscopy. *Scale bars* 0.2 μm (10 h), 0.5 μm (24 h), and 0.5 μm (72 h)

**Fig. 9:**
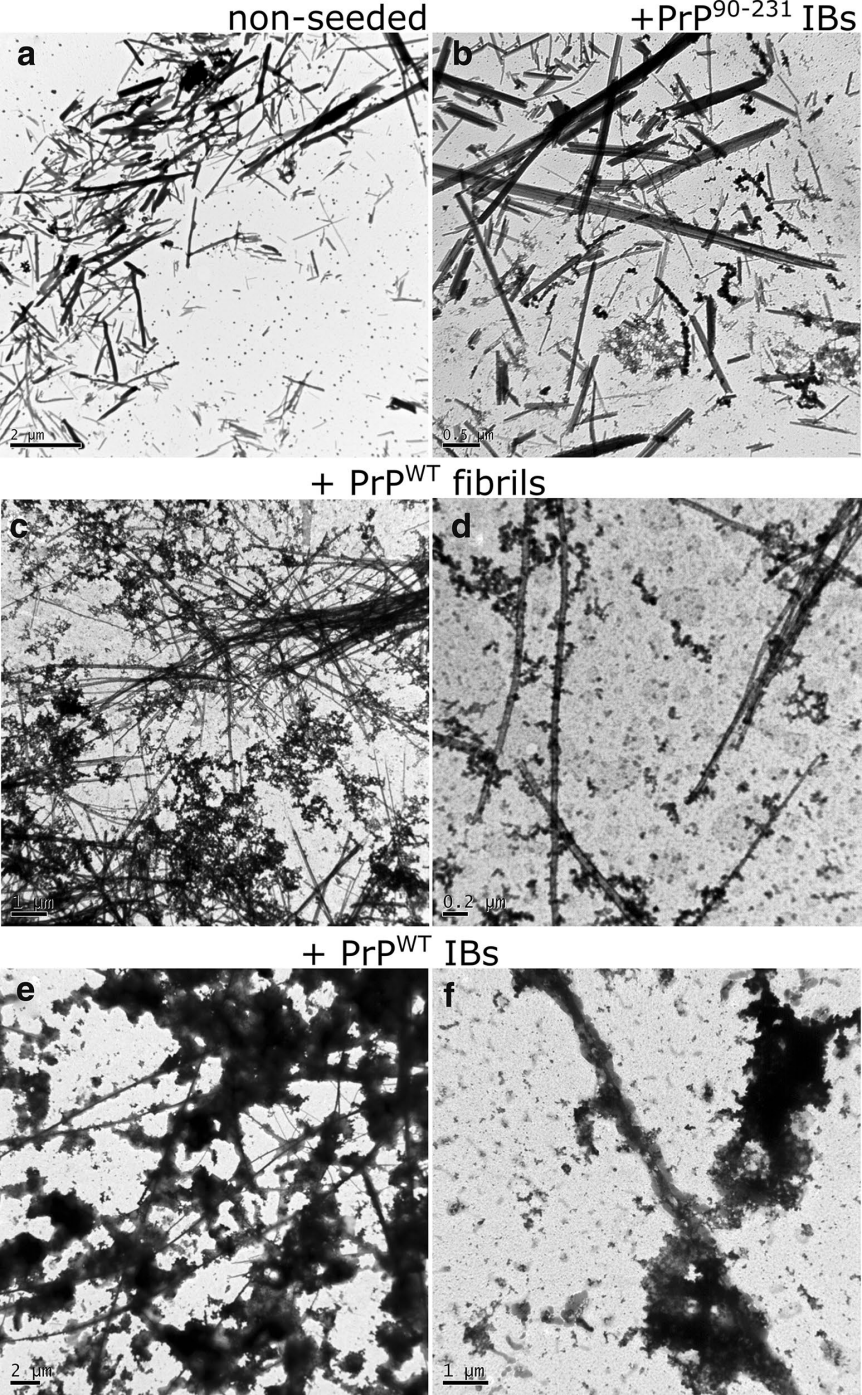
Morphology of PrP^WT^ aggregates at the end of the aggregation kinetics. Aggregates were collected at the end (~19 h) of non-seeded (**a**), seeded (**c**–**f**) and cross-seeded (**b**) reactions (Fig. 10) and they were monitored by negative staining and transmission electron microscopy. *Scale bars* 2 μm (**a**); 0.5 μm (**b**); 1.0 μm (**c**); 0.2 μm (**d**); 2 μm (**e**); 1 μm (**f**)

Transmission electron microscopy (TEM) was used to investigate the morphology of PrP^WT^ IBs and PrP^90–231^ IBs. Interestingly enough, we were able to observe amyloid fibrils in PrP^WT^ IBs, without any previous treatment with proteases or sonication, coexisting with less ordered aggregates (Fig. 7a, b). For the C-terminal PrP IBs (PrP^90–231^) we found uniformly shaped aggregates, likely corresponding to prefibrillar structures, similar to aggregates populated upon nucleic acid-induced PrP^WT^ aggregation (Fig. 7c, d) [36]. The different morphology of both aggregates fits well with their different stability and binding to amyloid dyes. The amyloid fibrils detected in PrP^WT^ IBs show lateral association and display a ribbon-like assembly composed of two or more aligned non-twisted flat filaments (Fig. 7b). These structural features resemble those of in vitro-formed amyloid fibrils by purified recombinant PrPs (see Fig. 8). In addition, we found also single filaments (Fig. 7a, b). This fibrillar disposition is normally found in in vitro-formed PrP amyloids [59]. The fibrils found in PrPWT IBs possess variable widths and may show some curvature, a feature of ribbon-like assemblies. Some ribbons were observed splitting apart either at their edges or at the middle, evidencing that they were still formed by individual protofibrils (Fig. 7b). We could also observe that some ribbons are associated with each other and form fibrils with a dichotomous pattern, a characteristic well described by Makarava et al. in 2006 for mouse PrP amyloid fibrils formed in vitro [58] (see Figs. 7a, b, 8, 9). This morphology was seen mostly during the nucleation phase of the fibril polymerization and was difficult to find during the subsequent elongation phase. Therefore, we conclude that this morphology represents a stage of early lateral association rather than dissociation of preformed fibrils into protofilaments [58]. In this same work, Makarava et al. showed well distinguishable subsets of fibrils formed by the hamster PrP under identic solvent conditions. It thus suggests that the murine PrP aggregates freely formed inside bacteria in our work can reproduce the same aggregation pathway in vitro without the need of a specific condition, such as buffer, pH, temperature and agitation. This polymorphism within fibrils has been attributed to the variable number of constitutive protofilaments and distinct modes of their lateral association within mature fibrils [58, 60]. In fact, scrapie fibrils derived from animals with prions diseases were also found to display high levels of polymorphism [11, 61, 62]. Thus, PrP^WT^ IBs constitute a bacterial reservoir of different PrP amyloid structures that coexist with more disordered aggregates all formed by the same sequence, exemplifying thus conformational diversity. The striking different morphology of PrP^90–231^ IBs and PrP^WT^ strongly suggests that the N-terminal PrP is a major contributor to the formation of the detected intracellular ordered amyloid-like assemblies.

### Amyloid seeding capacity of PrP^WT^ IBs

**Fig. 10:**
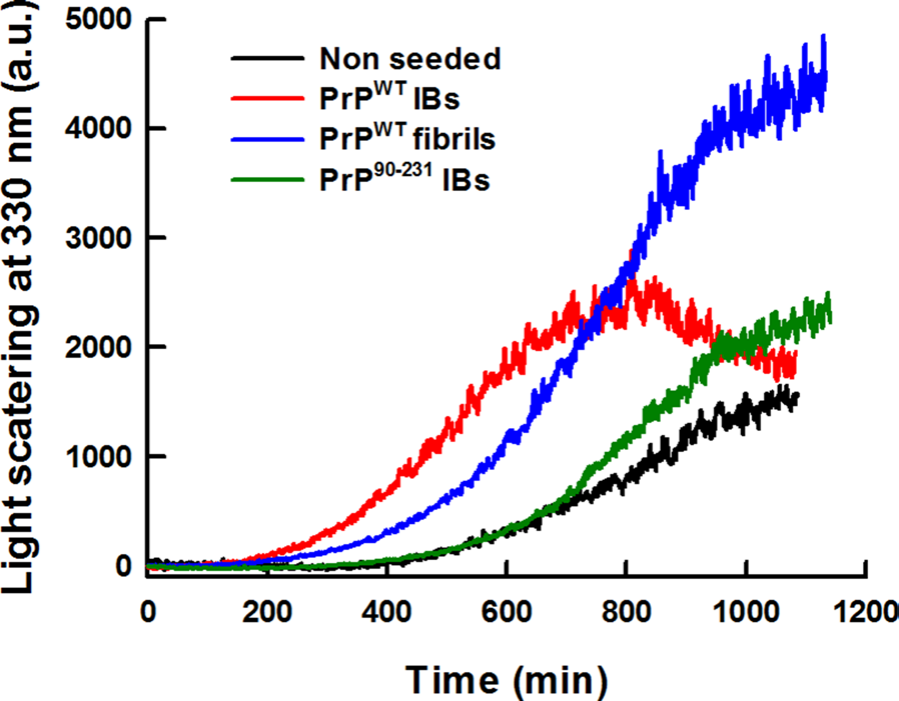
Aggregation kinetics of PrP^WT^. The aggregation reactions of 0.5 mg/mL of purified and refolded rPrP^WT^ were carried out under constant agitation at 600 RPM and 37 °C. In vitro preformed fibrils (2 %) or PrPs IBs (final OD_350_ = 0.1) were used for seeding and cross-seeding assays. PrP fibrilization (*black line*) as a function of time, exhibits a typical nucleation-elongation profile. The lag phase is reduced in the presence of pre-aggregated homologous protein, either PrP^WT^ fibrils (*blue line*) or PrP^WT^ IBs (*red line*). Cross-seeding with PrP^90–231^ IBs (*green line*) did not affect the fibrilization extent and kinetics

The kinetics of amyloid fibril formation usually follows a sigmoidal curve that reflects a nucleation-dependent growth mechanism [63]. As expected, the in vitro conversion of purified PrP^WT^ into fibrils follows this kinetic scheme (Fig. 8), with a lag phase of ~7 h, which corresponds to the formation of the initial nuclei and that is followed by polymerization or fibril growth, as confirmed by TEM imaging of the solution during the aggregation reaction (Fig. 8). Seeded protein aggregation is a well-established mechanism for in vivo amyloid fibril formation and underlies prion propagation [64, 65]. Accordingly, in the presence of 2 % preformed fibrils, the lag phase of the reaction is shortened to ~3.5 h. To test if the detected amyloid-like structures in PrP^WT^ IBs were able to template the conversion of its respective soluble species into amyloid fibrils, we performed the aggregation reaction in the presence of limited amounts of preformed purified PrP^WT^ IBs (Fig. 10). Interestingly enough, the effect exerted by IBs on fibril formation kinetics is similar to that promoted by the corresponding PrP^WT^ amyloid fibrils seeds, reducing the lag phase of the reaction to ~2 h. However, in PrP^WT^ IBs seeded reactions large aggregates that cannot be maintained in solution appear to accumulate after 12 h (720 min) of polymerization (Fig. 10). In contrast to amorphous aggregation, amyloid formation is a specific process that can only be seeded by sequentially and structurally homologous fibrils [66]. To evaluate if such selectivity also applies in the case of PrP IBs, we performed cross-seeding experiments, seeding the aggregation reaction of initially soluble PrP^WT^ with preformed and purified PrP^90–231^ IBs. Importantly, the presence of PrP^90–231^ IBs does not have any noticeable impact on the nucleation reaction, the overall kinetics resembling those of a non-seeded reaction (Fig. 10), thus confirming that a specific molecular recognition between soluble and fibrillar states is a requirement for seeding and that in the case of PrP^WT^ the N-terminal tail might play a crucial role in this process. Accordingly, it has been previously observed by high-pressure FTIR that the N-terminal domain modulates PrP misfolding and aggregation [67].

We analysed the morphology of the aggregates present in the final reaction of seeded and unseeded kinetics by TEM. In agreement with spectroscopic data, the presence of PrP^WT^ IBs and fibrils at the beginning of the reaction resulted in an increased number of amyloid fibrils in respect to non-seeded reactions or reactions seeded with PrP^90–231^ IBs. However, the acceleration of the fibrillation promoted by PrP^WT^ fibrils and especially by PrP^WT^ IBs results in the formation of apparently amorphous material tightly associated to the newly formed amyloid fibrils (Fig. 9). Interestingly, we could see after seeding PrP amyloid formation with PrP^WT^ IBs the presence of twisted fibrils (Fig. 9). Several early prion studies reported that filamentous structures were found in scrapie-infected rodent brain [68, 69]. In 1981, one report has called attention to helical fibrils formed by the twisting of two or four filamentous structures; they were found in preparations from brains of scrapie-infected rodents [70]. The ultrastructural morphology of these fibrils was reported to be different from that of many amyloids [71]. Nowadays, there are many reports providing information about the PrP twisted fibrillar structures [60].

Using small amounts of the PrP^WT^ IBs seeded solution for re-seeding PrP^WT^ soluble protein results in formation of visible fibrillar material that, when analysed by TEM, displays typical amyloid morphology coexisting with more amorphous material, confirming thus the striking ability of PrP^WT^ IBs to effectively propagate soluble protein conformational conversion into amyloid structures (Additional file 2).

### PrPs IBs display a proteinase K resistant core

**Fig. 11:**
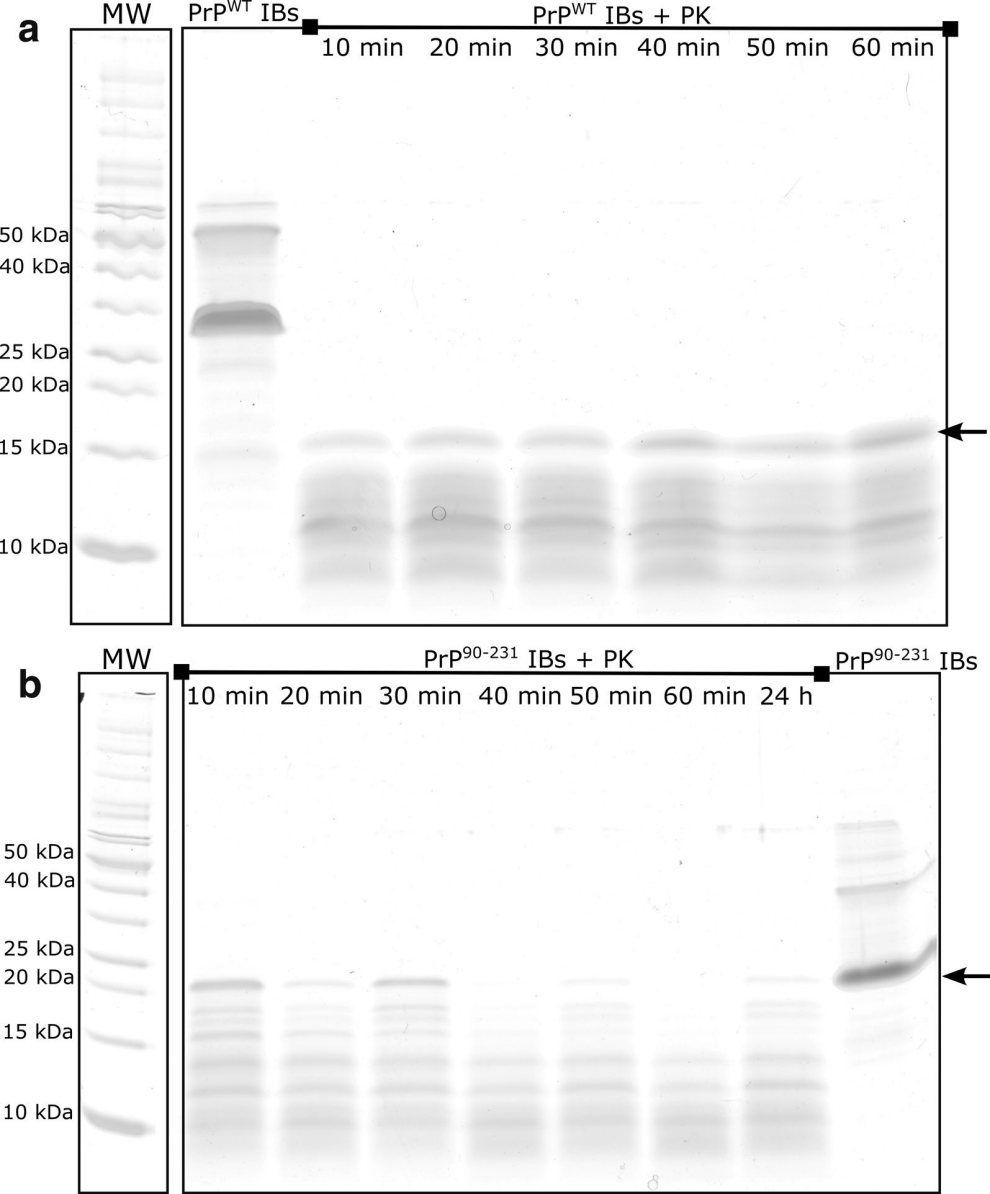
PK proteolysis of PrPs IBs. Tricine SDS-PAGE analysis of **a** PrP^WT^ IBs and **b** PrP^90–231^ IBs after incubation with 2.5 µg/mL PK at 37 °C for the indicated times. The smaller fragments remaining after PK proteolysis indicate a resistant core of these PrP IBs. The molecular weight ladder (MW) is placed offset for clarity. The *arrows* indicate C-terminal PrP fragments

Proteinase K (PK) is a protease normally used to map the protected core of amyloid fibrils. Despite its high activity for cleaving peptide bonds, PK cannot attack the highly packed backbones in an amyloid β-sheet structure. In contrast to soluble forms of PrPs, which are PK-sensitive, aggregated forms of PrP are known to have a PK-resistance profile that can be dependent on the in vitro aggregation conditions or even on the different in vivo sources from where they were extracted (such as diseased brains) [49, 72–75]. To verify the PK-resistance of the two different PrP aggregated deposits formed inside the cell, we evaluated the PK digestion of PrP IBs by tricine SDS-PAGE (Fig. 11). There were differences in the PK-digestion pattern between the two PrP IBs. Upon incubation of PrP^WT^ IBs with PK, the resistant fibrillar core is visualized as a major band with 16 kDa, which corresponds to the PrP C-terminal domain as evidenced by western blot analyses with the anti-PrP antibody R20 that recognizes a C-terminal epitope in PrP (residues 218–232) [76] (Additional file 3). This fragment possesses the same apparent molecular weight of the C-terminal domain (residues 90–231) (16 kDa), indicating that the N-terminal domain was cleaved and the rest of the protein was in a protected conformation. Most of the resultant fragments of PK-digested PrP^WT^ IBs, which vary from ~15 to 6 kDa were labeled by R20, and even after 60 min of reaction the larger fragment (16 kDa) persists (Fig. 11a). Indeed, the PK-resistant core of the pathogenic PrP^Sc^ corresponds also to the PrP C-terminal domain [14]. The PK resistance of PrP^WT^ IBs is comparable with the PK-resistant form of PrP^Sc^. In PrP amyloid IBs we studied here, the 16 kDa band resisted PK treatment under conditions that are commonly used for PK reactions with the scrapie brain homogenates (incubation for 1 h at neutral pH) [77, 78]. In addition, this same resistant fragment (16 kDa) is also observed upon PK-digestion of the in vitro-formed PrP amyloid fibrils [79]. In contrast, PK-digestion of PrP^90–231^ IBs showed a different profile; after 30 min of PK-treatment the apparent 16 kDa fragment was completely degraded, and the remaining resistant fragments have lower molecular mass (Fig. 11b). These PrP IBs do not possess the same conformation and, thus, as expected, differ in their biochemical and biophysical properties. Recently, it was shown that full-length recombinant PrP (23–231) can aggregate into a β-sheet-rich oligomeric species (~12 PrP molecules), and that the protein N-terminal region (23–90) was necessary for the formation of this oligomer [80]. At the same condition, PrP lacking part of the N-terminal region (PrP91-231) aggregated into heterogeneous species [80]. It is increasingly recognized the essential role of the N-terminal domain in directing PrP intermolecular association and in promoting the stability and proper folding of the C-terminal domain [80–82]. Taken together, this result is in good agreement with the lower stability we have shown to extracted PrP^90–231^ IBs relative to PrP^WT^ IBs (Fig. 4).

**Fig. 12:**
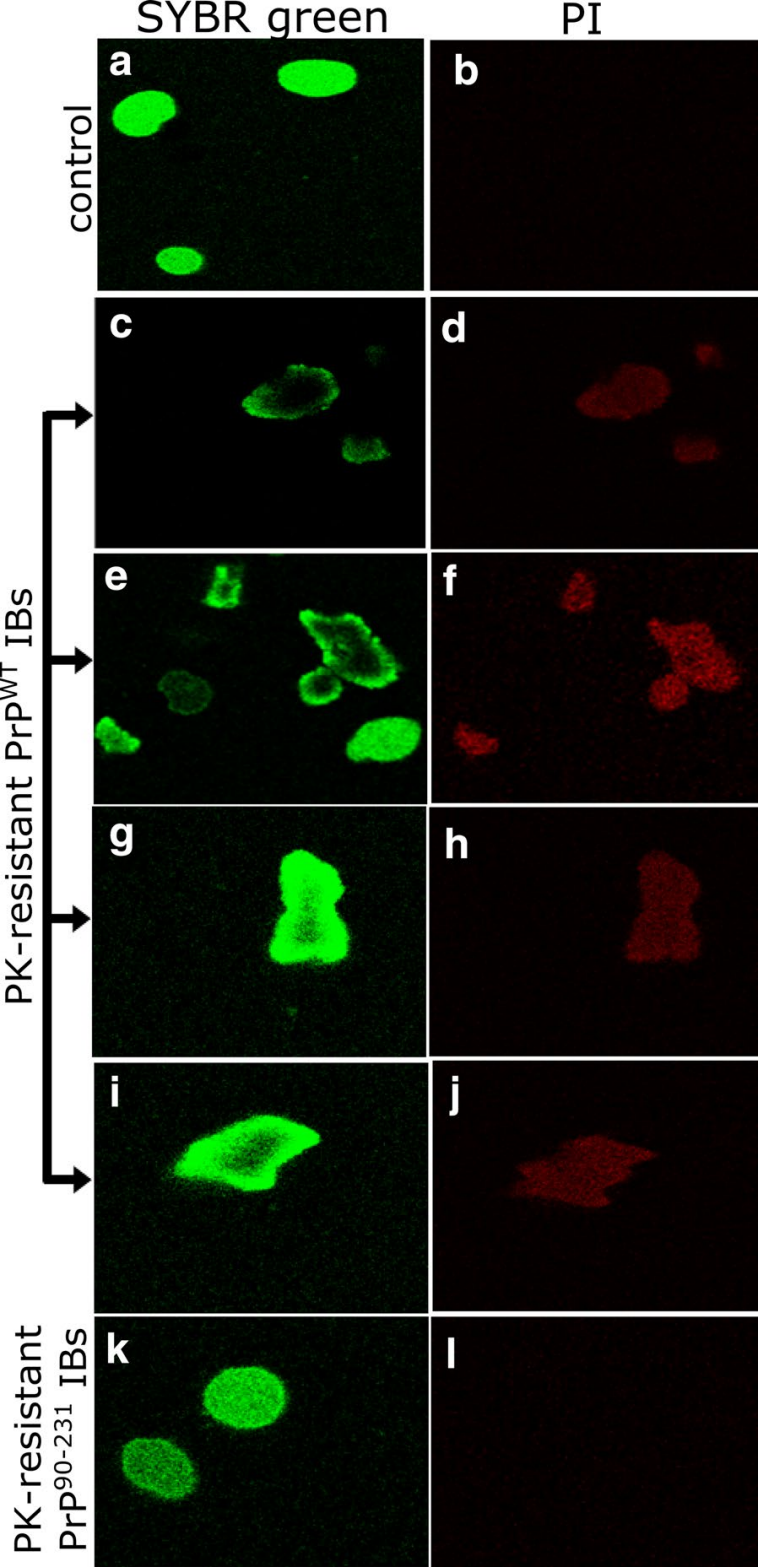
Cytotoxicity of the PK-resistant core of PrP IBs visualized by confocal microscopy. Confocal fluorescence microscopy images of SH-SY5Y cells stained with SYBR *green* or propidium iodide (PI). Cells were treated with PK-derived fragments of PrP^WT^ IBs (*panels* **c**–**j**), or PrP^90−231^ IBs (**k**, **l**). All samples were incubated with PK for 1 h at 37 °C and PK was inactivated before applying to cells. Inactivated PK in PBS was used as control (**a**, **b**). Representative images of two independent experiments done in duplicate are shown. Further details are described in the “Methods”

To confirm the conformational diversity exhibited by PrP^90–231^ and PrP^WT^ we explored whether their differently protected amyloid cores display different cytotoxic properties. To this aim PrP IBs where digested with PK for 60 min, the protease was inactivated and the digested aggregates were administered to neuroblastoma SH-SY5Y cells. The combination of SYBR green and propidium iodide (PI) staining allows assessing the cell viability by confocal fluorescence microscopy, as viable cells are permeable to SYBR green and PI only enters cells with permeabilized membranes corresponding to dead cells. The impact of aggregates in cell morphology was also monitored (Fig. 12). Cells treated with inactivated PK were used as negative controls. Cells treated with the PK-resistant PrP^90–231^ core exhibited normal morphology and were not stained with PI, indicating that it does not exert significant toxicity. In contrast, the PK-resistant PrP^WT^ core was neurotoxic, since its presence resulted in cell death (cells were stained with PI) and in abnormal cell morphology. Although the prion infectious region is attributed to the resistant 90–231 core (PrP27-30 in vivo), we did not see neurotoxic activity for PK-digested PrP^90–231^ IBs, only for digested PrP^WT^ IBs. We showed here that the protease-resistant region of the PrP^WT^ IBs, after 60 min of reaction, belongs to the C-terminal domain in contrast to PrP^90–231^ IBs digestion, which renders smaller fragments that do not bind to PrP antibody (R20) (Additional File 3). One might speculate that the largest fragment of the C-terminal resistant to PK-digestion (the 16 kDa fragment) is necessary to retain cytotoxicity; this fragment might have its intact disulphide bridge which is well known to be necessary to cause prion diseases [83]. Further experiments will be required to address which are the residues involved in the toxic conformations we have seen for these PrP^WT^ IBs PK-resistant core. Therefore, they are not the same species, and thus, are expected to result in different cytotoxic effects. Probably, the low molecular weight fragments derived from PrP^90–231^ IBs are not properly folded into a toxic conformation. Moreover, the amount of PK-resistant fragments for both PrP constructs differs, as the sensitivity to PK is depends on the aggregated structure.

## Conclusions

Prions are misfolded, self-propagating and infectious proteins. The bacterial inclusion bodies formed by fungal prions, such as HET-s PFD, Ure2p and Sup35-NM have been shown to display an amyloid fold and to be infective. This ability to embrace potentially harmful misfolded polypeptides into insoluble deposits seems to be a strategy mechanism perpetuated along the evolution from prokaryotic bacteria to highly complex eukaryotic organisms. We showed here for the first time that the IBs formed by mammalian prion proteins are also enriched in seeding competent amyloid-like structures, supporting the formation of prion-like conformations inside bacteria. Moreover, these aggregates display conformational diversity, thus becoming an interesting and simple model to study how this property can be modulated in vivo by the quality control machinery. Since PrP accumulates in IBs at high levels and these biological particles are easily purified, it is suggested that they might become a convenient source to obtain prion particles. It is clear that the bacterial cytosol where these aggregates are formed differs from that of eukaryotic cells; however, the potentiality of these inside-the-cell formed amyloid particles to adopt infective conformations is, in our opinion, much higher than the one of the aggregates formed by the purified recombinant protein in vitro after complete denaturation and refolding procedures. In addition, as already shown for other amyloids [15], PrP-producing bacterial cells can potentially be used for the easy and cheap screening of anti-aggregation compounds able to prevent intracellular PrP amyloid-like aggregation, being thus useful in the early stages of discovery of anti-prionic drugs.

## Methods

### Prion proteins expression and purification

*E. coli* C43 (DE3) cells were transformed with plasmids (pET-28 b) encoding the murine wild-type prion protein (PrP^WT^) encompassing residues 23–231 and the murine PrP C-terminal domain residues 90–231 (PrP^90–231^), both containing a histidine-tag. Cells were grown aerobically in liquid Luria–Bertani (LB) medium containing appropriate antibiotics in a rotary shaker at 37 °C and 250 RPM. Overnight cultures were diluted 100-fold in LB and allowed to grow to an OD_600_ = 0.7. At OD_600_ = 0.7, expression was induced with 1 mM isopropyl-1-thio-β-d-galactopyranoside (IPTG). Cells were harvested after overnight induction (18–20 h), centrifuged, resuspended in 60 mL of buffer U (8 M urea, 10 mM Tris–HCl, 100 mM NaH_2_PO_4_, 10 mM reduced glutathione, pH 8.0). After sonication and centrifugation, the soluble protein fraction was added to 5 mL of HisTrap FF prepacked column (GE Healthcare) and washed with 30 mL of buffer U. On-column oxidative refolding was performed by applying for 2 h a 160 mL-gradient of buffer U to buffer B (10 mM Tris–HCl, 100 mM NaH_2_PO_4_, pH 8.0). Then, the column was washed with 50 mL buffer B. Unspecific bound proteins were removed from the column with 50 mL of 50 mM imidazole in buffer B. The recombinant proteins PrP^WT^ and PrP^90–231^ were eluted with buffer E (10 mM Tris, 100 mM NaH_2_PO_4_, 750 mM imidazole, pH 5.8). Histidine tail-fused PrP was dialyzed against milliQ water at least two times. The histidine tail was removed from the prion protein using thrombin (1:1000). The cleavage reaction was carried out at room temperature for 2 h. After thrombin cleavage, the sample was repurified in the HisTrap column. Finally, the protein solution was dialyzed against milliQ water two times to remove any remaining salt. 15 % SDS-PAGE analysis revealed more than 95 % of purity.

### PrP^WT^ and PrP^90–231^ IBs extraction

IBs were purified from IPTG-induced cells harbouring the pET-28(b)/PrP^WT^ plasmid, the pET-28(b)/PrP^90–231^ plasmid and vector alone by detergent-based procedures. IBs were purified from induced cell extracts by detergent-based procedures as previously described [17]. Briefly, cells in a 10 mL culture were harvested by centrifugation at 12,000*g* (at 4 °C) for 15 min and resuspended in 200 µL of lysis buffer (50 mM Tris–HCl pH 8.0, 1 mM EDTA, 100 mM NaCl), plus 30 µL of 100 mM protease inhibitor PMSF and 6 µL of a 10 mg/mL lysozyme solution. After 30 min of incubation at 37 °C under gentle agitation, Nonidet-P40 was added at 1 % (v/v) and the mixture was incubated at 4 °C for 30 min. Then, DNase I and RNase were added to a final concentration of 25 μg/mL and 3 µL of 1 M MgSO_4_ was added. The resulting mixture was further incubated at 37 °C for 30 min. Protein aggregates were separated by centrifugation at 12,000*g* at 4 °C for 15 min. Finally, IBs were washed once with the same buffer containing 0.5 % Triton X-100 and once with phosphate buffered saline (PBS). After a final centrifugation at 12,000*g* for 15 min, pellets were stored at −20 °C until analysis. The frozen pellets were reconstituted in PBS. SDS-PAGE analysis revealed that in all cases the murine prion proteins were the major polypeptidic components of the aggregates. Prion proteins concentration in IBs was estimated using image densitometry software ImageJ in the SDS-PAGE gel analysis in comparison with the respective dosed purified protein. We performed the same procedures with cells extracts of bacteria containing an empty plasmid as control IBs.

### Secondary structure determination

Attenuated total reflectance (ATR)-Fourier Transformed Infrared spectroscopy analyses of PrP^WT^ and PrP^90–231^ IBs were performed using a Nicolet 6700 IR spectrometer (Thermo Scientific, USA) equipped with an ATR accessory. Dried samples were applied directly to the ATR crystal to be analysed. Each spectrum consisted of 128 accumulated scans, measured at a spectral resolution of 4 cm^−1^ within the mid-IR range (4000–675 cm^−1^). Fourier deconvolution of the FTIR spectra was performed with a resolution enhancement factor of 1.6 and a bandwidth of 21 cm^−1^. Peak position and curve fitting were determined with OMNIC™ software v. 8.0 (Thermo Scientific WI, USA) with a mixed Gaussian-Lorenztian function, allowing assignment of different secondary structure components in the amide I range (1700–1600 cm^−1^) [47, 48, 67].

### Congo red binding

To get insights into the amyloid nature of the PrP^WT^ and PrP^90–231^ IBs, CR binding assays were performed. The interaction of 20 μM CR with the purified IBs (final OD_600_: 0.1) was tested using a Cary100 UV/Vis spectrophotometer (Varian, Palo Alto, CA, USA). CR binding was quantified by the equation: CR bound = Abs_540nm_/25,295 − ABS_477nm_/46,306 [84]. The extent of amyloid structure was measured by the increase of CR bound to PrP IBs in relation to control IBs [IBs purified from IPTG-induced cells harbouring only the pET-28(b) plasmid].

### Thioflavin T (Th-T) binding assay

Th-T binding was used to probe amyloid presence in the samples. Incubation of 30 μM Th-T with PrP^WT^ IBs and PrP^90–231^ IBs (final OD_600_: 0.1) or the correspondent amyloid fibrils was recorded using a Jasco FP-8200 spectrofluorometer (Jasco Inc, MD, USA) with an excitation wavelength of 445 nm and emission range from 480 to 580 nm at 37 °C in PBS. Five individual scans were averaged for each measurement. The intensity of the spectra at the 482 nm maximum was recorded as an indication of the extent of amyloid conformation in the aggregates.

### Thioflavin-S binding in living cells

Detection of thioflavin-S (Th-S) binding was performed in living cells expressing PrP^WT^, or PrP^90–231^ and control non-induced cells. Bacterial cells were washed with PBS buffer and diluted at an OD_600_ of 0.1. Th-S was added at 125 µM final concentration; cells were then incubated for 1 h and washed twice with PBS. Cells were placed on top of a microscope slide and covered with a cover slip. Photographs were acquired at 40-fold magnification under UV light in a Leica fluorescence microscope (Leica DMRB, Heidelberg, Germany).

### Chemical denaturation

For stability assays, purified PrP^WT^ and PrP^90–231^ IBs were prepared at final OD_350_ = 1 in PBS containing selected concentrations of urea ranging from 0 to 8 M. The reactions were allowed to reach equilibrium by incubating them for 12 h at room temperature. The fraction of soluble protein (f_S_) was calculated from the fitted values using equation: f_S_ = 1 − ((y_S_ − y)/(y_S_ − y_A_)), where y_S_ and y_A_ are the absorbance at 350 nm of the soluble and aggregated protein, respectively, and *y* is the absorbance of the protein solution as a function of the denaturant concentration. The value m_1/2_ was calculated as the denaturant concentration at which f_S_ = 1/2. OD_350_ changes were monitored with a Cary400 Varian spectrophotometer.

### Transmission electron microscopy (TEM)

Each sample (20 μL) was applied to a carbon coated copper grid, and after 5 min the grid was washed with MilliQ water. Samples were stained with 2 % (w/v) uranyl acetate for 1 min and then washed again. Images were collected on a Jeol 1200 microscope (Boston, MA, USA) operating at 80 kV.

### In vitro conversion of PrP into amyloid fibrils

To target amyloid fibril formation, PrP solutions were prepared immediately before use by resuspending lyophilized purified PrP^WT^ powder in 4 M GdnHCl, 0.02 M thiourea, and 0.1 M MES, pH 6.0, in a protocol adapted from previous studies [59]. Samples were centrifuged at 12,000*g* for 5 min and the protein concentration was determined by its extinction coefficient at 280 nm (63,495 M^−1^ cm^−1^), calculated from the PrP^WT^ primary sequence in http://web.expasy.org/protparam/. The fibrillation reactions of 0.5 mg/mL PrP were carried out in 1.5-ml conical low-binding plastic tubes up to a total reaction volume of 0.6 ml at 37 °C with continuous shaking at 600 rpm for at least 3 days using an Eppendorf Thermomixer Comfort (Eppendorf, USA). Aliquots from each sample were taken over time.

### Seeding assays

PrP aggregation departing from monomeric recombinant PrP was monitored by measuring the transition from non-aggregated to aggregated state by following light scattering at 350 nm in a Jasco FP-8200 spectrofluorometer (Jasco Inc, MD, USA). The polymerization reactions showed typical nucleation-elongation kinetics of amyloid formation. The reactions were carried out with 0.5 mg/mL of soluble purified PrP^WT^ in 4 M GdnHCl, 0.02 M thiourea, and 0.1 M MES pH 6.0 using 1 cm-path length quartz cuvette in a total reaction volume of 1 mL at 37 °C with continuous shaking at 600 rpm using micro-stir bars. In the seeding assays, a solution of PrP^WT^ IBs resuspended in PBS with OD_350_ of 10.0 were sonicated for 10 min, and then diluted 100-fold (final OD_350_ = 0.1) at the beginning of the reaction. The seeding ability of 2 % preformed fibrils (after 10 min of sonication) was also evaluated. Cross-seeding assays were performed in the same manner by adding a sonicated solution of PrP^90–231^ IBs (final OD_350_ = 0.1) to initially soluble PrP^WT^.

### Confocal microscopy and image processing

Confocal images of human neuroblastoma (SH-SY5Y) cell cultures were captured in complete medium at 37 °C, using a laser scanning confocal microscope (Leica TCS SP2 AOBS equipped with a HCX PL APO 63 × 1.4 oil, immersion objective, Germany). Briefly, SH-SY5Y cells were seeded in 35 cm^2^ plates (Mat Tek) with approximately 30 % of confluence in complete medium and incubated for 72 h in the presence of sterile PBS buffer + PK (positive control) and the PK-resistant core of the PrPs IBs. Proteinase K was inactivated by boiling all solutions before applying them to cultured cells. Cells were incubated with 0.5 μg/mL SYTO green and 10 μg/mL propidium iodide (PI) (Molecular Probes) for 15 min at 37 °C and washed twice with PBS buffer. Cell morphology was analysed by confocal fluorescence microscopy using an orange diode (588–715 nm emission collected) and a UV laser (excited at 350 nm and collected at 405 nm). Two independent experiments, both in duplicate were done and the entire field of each plate was observed at the microscope.

### Proteinase K (PK) resistance assay

The PK concentration in this assay was optimized in preliminary experiments (not shown). PrP^WT^ IBs and PrP^90–231^ IBs at final OD_350_ of 0.5 were incubated with PK (Sigma-Aldrich, USA) at final concentration of 2.5 μg/mL in PBS for 1 h at 37 °C. Aliquots of PK digestion were taken at every 10 min and the reaction quenched by the addition of the same amount of 4 times concentrated denaturing sample buffer. Samples were heated at 95 °C for 5 min and analysed by Tris–Glycine SDS-PAGE. The assay with soluble purified recombinant PrP was performed in the same manner [85].

## Additional files

10.1186/s12934-015-0361-y In the Supplemental Material Section the Amide I region of the ATRFTIR spectrum of purified native recombinant Pr^P23–231^ is shown, along with the corresponding spectral bands assigned to different secondary structure components.
10.1186/s12934-015-0361-y In the Supplemental Material Section results from visual and TEM observation of fibrils formed after a second consecutive round of PrP^WT^ aggregation using PrP^WT^ IBs as seeds are presented.
10.1186/s12934-015-0361-y In the Supplemental Material Section results from western blot analyses of PrP^WT^ IBs and PrP^90–231^ IBs after PK-digestion are presented.
