# Supplementary material for: Mammalian prion protein (PrP) forms conformationally different amyloid intracellular aggregates in bacteria
Source: Microb Cell Fact. 2015 Nov 4;14:174. doi: 10.1186/s12934-015-0361-y (PMC4634817; doi:10.1186/s12934-015-0361-y)
Supplement: Supplementary file 1 — 10.1186/s12934-015-0361-y In the Supplemental Material Section the Amide I region of the ATRFTIR spectrum of purified native recombinant PrP23–231 is shown, along with the corresponding spectral bands assigned to different secondary structure components. [file 12934_2015_361_MOESM1_ESM.docx]

**Additional file 1. Secondary structure content of purified native recombinant PrP (rPrP^23-231^).** ATR-FTIR spectrum (solid black line) of a dry sample of purified recombinant full-length murine PrP was further processed with OMNIC^TM^ software (see Methods) and spectral components in the Fourier deconvoluted FTIR amide I region (dashed gray line) are shown as bands 1 to 5 (solid gray lines). The assignement to different structural components is as follows: 1 (1689 cm^-1^): turns/β-sheet; 2 (1678 cm^-1^): turns; 3 (1653 cm^-1^): α-helix/random coil; 4 (1629 cm^-1^): β-sheet; 5 (1616 cm^-1^): β-sheet.

**
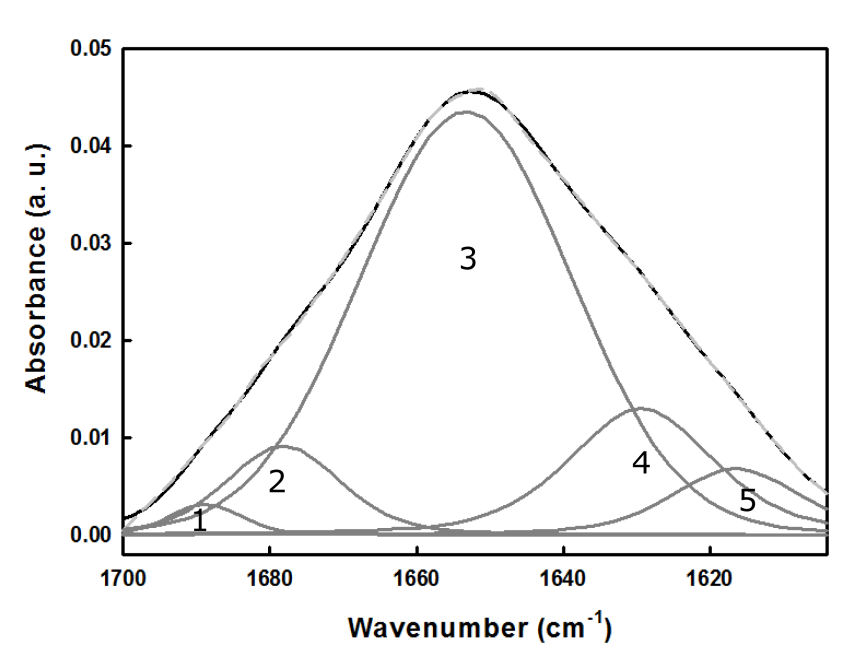
**
