# Supplementary material for: Mammalian prion protein (PrP) forms conformationally different amyloid intracellular aggregates in bacteria
Source: Microb Cell Fact. 2015 Nov 4;14:174. doi: 10.1186/s12934-015-0361-y (PMC4634817; doi:10.1186/s12934-015-0361-y)

**Additional file 2. Second consecutive round of seeding of PrP^WT^ polymerization with PrP^WT^ IBs.** Re-seeding soluble PrP^WT^ with 10 % of fibrils formed after a first seeding reaction with PrP^WT^ IBs promotes a strong and fast aggregation reaction resulting in fibrillar structures as visualized by eye (A) displaying typical amyloid features as observed by TEM (B).


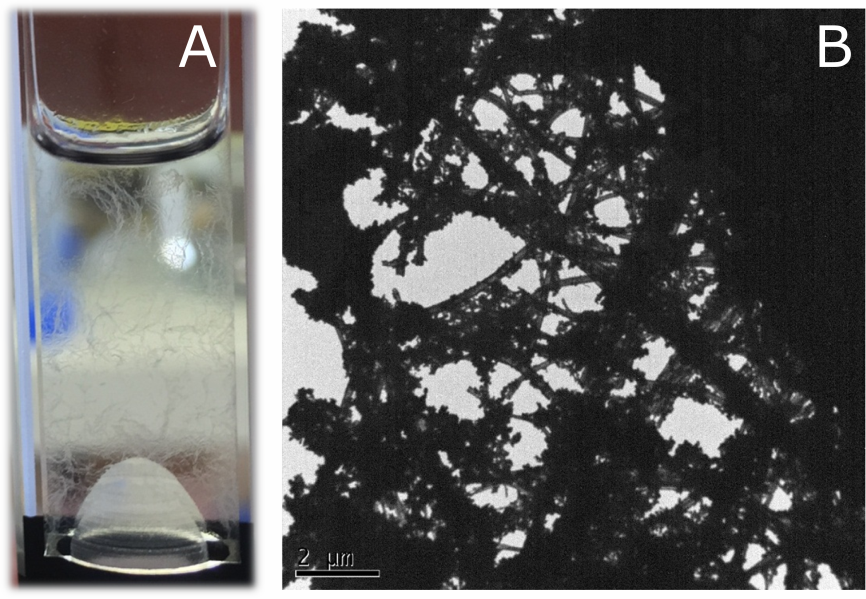

Supplement: Supplementary file 2 — 10.1186/s12934-015-0361-y In the Supplemental Material Section results from visual and TEM observation of fibrils formed after a second consecutive round of PrPWT aggregation using PrPWT IBs as seeds are presented. [file 12934_2015_361_MOESM2_ESM.docx]
