# Supplementary material for: Mammalian prion protein (PrP) forms conformationally different amyloid intracellular aggregates in bacteria
Source: Microb Cell Fact. 2015 Nov 4;14:174. doi: 10.1186/s12934-015-0361-y (PMC4634817; doi:10.1186/s12934-015-0361-y)

**Additional file 3. Western blotting analyses of PrP^WT^ IBs and PrP^90-231^ IBs after PK-digestion.** PrP^WT^ IBs (A) and PrP^90-231^ IBs (B) were digested with 2.5 mg/mL of proteinase K (PK) for 10 min or 60 min at 37 °C in PBS. The reaction was stopped by the addition of denaturing buffer. Samples were resolved by 12.5 %-SDS-PAGE for 40 min at 180 volts, and then transferred to a polyvinylidene difluoride (PVDF) membrane (Bio-Rad Laboratories, Mississauga, ON). Membranes were blocked overnight in the Odyssey blocking buffer. Then the membranes were probed for 4 hours with primary antibody anti-PrP (R20) (1:5,000 dilution) [see reference 76] that recognizes a C-terminal epitope in PrP (residues 218–232). The membrane was then washed 5 times in PBS with 0.1 % Tween-20 (PBST) for 5 min each wash. After that the membranes were incubated for 1 hour with the anti-rabbit secondary fluorescent antibody IRDye 800CW (diluted 1:15,000 in PBST). Thereafter the membranes were washed three times with PBST prior to be detected in the 800 channel of Odyssey® Imaging Systems.


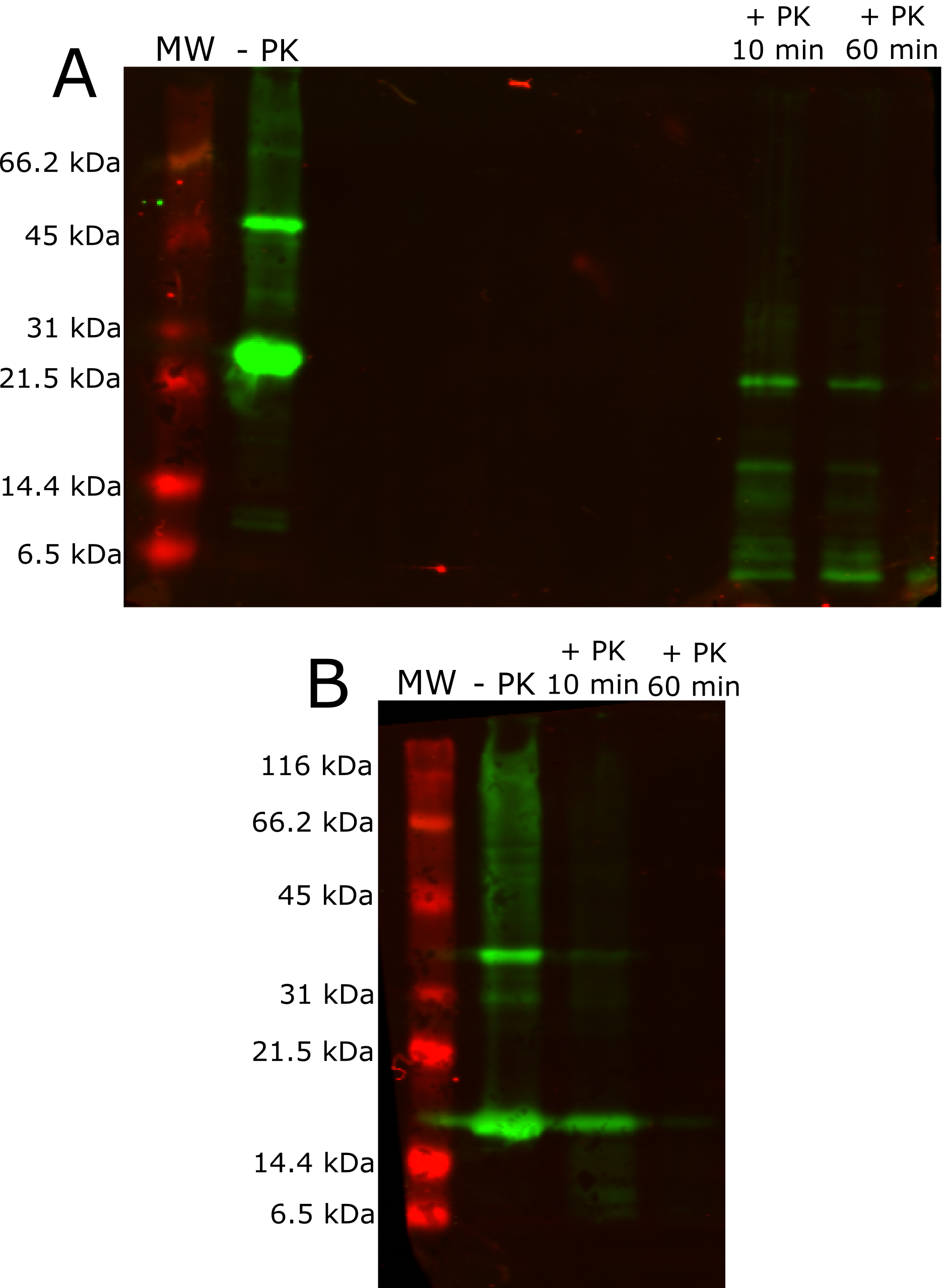

Supplement: Supplementary file 3 — 10.1186/s12934-015-0361-y In the Supplemental Material Section results from western blot analyses of PrPWT IBs and PrP90–231 IBs after PK-digestion are presented. [file 12934_2015_361_MOESM3_ESM.docx]
